# Protective Effect of *Prunus mume* Fermented with Mixed Lactic Acid Bacteria in Dextran Sodium Sulfate-Induced Colitis

**DOI:** 10.3390/foods10010058

**Published:** 2020-12-29

**Authors:** Jeong-Ho Kim, Yeong-Seon Won, Hyun-Dong Cho, Seong-Min Hong, Kwang-Deog Moon, Kwon-Il Seo

**Affiliations:** 1 Department of Food Science and Technology, Kyungpook National University, Daegu 41566, Korea; kimjeoho90@gmail.com (J.-H.K.); kdmoon@knu.ac.kr (K.-D.M.); 2 Department of Food Biotechnology, Dong-A University, Busan 49315, Korea; wonys@dau.ac.kr; 3 Department of Pharmacology & Regenerative Medicine, University of Illinois, Chicago, IL 60612, USA; chd0811@hanmail.net; 4 College of Pharmacy and Gachon Institute of Pharmaceutical Science, Gachon University, Incheon 21936, Korea; homsgm0517@gmail.com

**Keywords:** colitis, fermentation, lactic acid bacteria, pro-inflammatory cytokine, *Prunus mume*

## Abstract

The fruit of *Prunus mume* (PM) is widely cultivated in East Asia, and it has been used as a folk medication for gastrointestinal disorders, e.g., diarrhea, stomach ache and ulceration. In this study, the pectinase-treated PM juice (PJ) was fermented with *Lactobacillus* strains containing fundamental organic acids and free amino acids. The PJ fermented with *Lactobacillus plantarum* and *L. casei* (FP) was investigated for its protective effect in dextran sodium sulfate (DSS)-induced colitis mice model. The administration of FP reduced lipid peroxidation and histopathological colitis symptoms, e.g., shortening of the colon length, depletion of mucin, epithelial injury and ulceration, in colonic tissues. The FP-supplemented group showed the alleviation of pro-inflammatory cytokines. Compared with the DSS control group, the supplementation of FP significantly reduced the levels of serum interferon-γ (IFN-γ), interleukin (IL)-1β, IL-6, IL-12 and IL-17 as well as colonic tumor necrosis factor-α, IFN-γ, IL-12 and IL-17. Furthermore, the DSS-induced TUNEL-positive area was significantly reduced by the FP supplementation. These results show that the supplementation of FP fermented with mixed lactic acid bacteria, *L. plantarum* and *L. casei*, elucidated the protective effect in DSS-induced colitis mice. Hence, this study suggests that FP can be utilized as a natural therapeutic agent for colitis and intestinal inflammation.

## 1. Introduction

Inflammatory bowel disease (IBD), a chronic disease, includes ulcerative colitis (UC) and Crohn’s disease [1]. UC, a type of inflammatory bowel disease, is a chronic disease that affects both genders [2]. The disease occurs in the rectal and sigmoid colon and causes a decrease in body weight, diarrhea with bloody excrement and shortening of the colon length [3]. Researchers have focused on discovering the molecular mechanisms for the occurrences of this disease and identifying new therapeutic possibilities without the addition of corticosteroids, which can cause severe side effects e.g., diabetes, osteoporosis and headache [4,5]. Dextran sodium sulfate (DSS), a sulfated polysaccharide with an anticoagulant, -induced colitis mouse model is used widely for investigating the effects of anti-colitis therapeutic agents and natural products because aspects of the disease pathology and immune response resemble human UC.

*Prunus mume* fruit, also as known as maesil, meizi and ume, containing fundamental organic acids such as citric acid and malic acid, as well as various phenolic compounds, e.g., chlorogenic acid derivatives and 5-HMF, is cultivated widely in East Asia [6,7,8]. It has been used as a folk medication for thirst, vomiting, fever, digestion and gastrointestinal disorders [9,10]. The inhibitory effects of *Prunus mume* on intestinal inflammation have been reported for a decade. The *Prunus mume* extract decreased pro-inflammatory cytokines, which showed high radical scavenging and superoxide dismutase-like activities [11]. In a rat model of chronic cerebral hypoperfusion, the ethanol extract of *Prunus mume* ameliorated neurological dysfunction by inhibition of pro-inflammatory cytokines [12]. Further, Lee et al. (2017) [13] reported that *Prunus mume* extract showed an anti-colitis effect on DSS-induced colitis mice. However, the effect of LAB-fermented *Prunus mume* juice (PJ) to improve the features of colitis in mice needs to be explored.

Fermentation, which is one of the ancient methods of a processing method for food preservation globally, is a technology using microorganisms and their growth and metabolic activities in foods, leading to enhanced food qualities [14,15]. It is also a comparatively cost-effective and low-energy process [16]. Lactic acid bacteria (LAB) are one of the most popular microorganisms in food fermentation and industrial food applications because of their physiological characteristics such as high acid tolerance and broad growth temperatures ranging from 20 to 45 °C [17]. Many resources fermented by LAB show improved functionality. Lizardo et al. (2020) [18] reported that the phytochemical content and antioxidant activities of cherry silverberry fruit was increased by fermentation with *Lactobacillus plantarum* KCTC 33131 and *L. casei* KCTC 13086. Khan et al. (2018) [19] reported that LAB-fermented dried longan contained higher phenolic and flavonoid contents and antioxidant capacities compared to unfermented dried longan. Pharmacological studies of LAB have been reported to convince the improvement of therapeutic effects, e.g., diarrhea, constipation and colitis [20,21,22]. Furthermore, LAB co-cultures could produce added value according to the increase in food quality, such as flavor and texture, and enhance functionality [23].

Therefore, in this study, the fermented *Prunus mume* juice with *L. plantarum* and *L. casei* (FP) containing varied free amino acids and organic acids was produced, and the physicochemical characteristics were investigated. Moreover, the protective effect of FP was evaluated by the colitis-related markers on DSS-induced colitis mice model.

## 2. Material and Methods

### 2.1. Materials 

PJ used for production of FP was prepared by the method of Cho et al. (2019) [24]. Briefly, *P. mume* fruits purchased from the Korea Maesil Organization (Suncheon, Korea) were thoroughly washed with tap water, crushed and reacted with 0.1% (*w*/*v*) pectinase (Pectinex Ultra AFP, Novozyme, Switzerland, 10,000 Pectu/g) at 40 °C for 2 h. After that, the samples were centrifuged at 3500× *g* for 15 min at 4 °C. The supernatant was filtered and concentrated using rotary evaporator until 56–60 °Brix was reached. *Lactobacillus plantarum* KCTC 33131 and *L. casei* KCTC 13086 were obtained from the Korea Collection for Type Cultures (Jeongeup, Korea).

### 2.2. Production of FP

LAB-fermentation using the PJ was conducted three times by batch culture. An amount of 2.5 g of PJ was adjusted to 100 mL with distilled water (DW), and mono- and mixed cultures of *L. plantarum* and *L. casei* (2%, *v*/*v*) were inoculated into them (Table 1). Fermentation was accomplished in shaking incubator at 37 °C and 150 rpm for 72 h.

### 2.3. Total Soluble Solids, pH and Cell Counting

The changes of total soluble solids and pH of samples were measured every 24 h for fermentation time. The changes of LAB population during fermentation were determined every 24 h by standard plate count method.

### 2.4. Organic Acid and Free Amino Acid Contents

The analysis of organic acid contents in FP were determined by high-performance liquid chromatography (Shimadzu Co. Model Prominence, Kyoto, Japan) as previously described [8]. Briefly, the separation of organic acids was conducted using a PL Hi-Plex H column (7.7 × 300 m^2^ Agilent Co., Santa Clara, CA, USA) at 65 °C. The mobile phase consisted of 5 mM sulfuric acid with a flow rate of 0.6 mL/min. The chromatographic peak coinciding with each organic acid was identified by comparing the retention time with that of each standard.

The analysis of free amino acid contents in FP was carried out using an amino acid autoanalyzer (L-8900, Hitachi, Tokyo, Japan) with Hitachi custom ion exchange resin (2622 SC PF, 4.6 × 60 m^2^) as previously described [8]. The column was maintained at 50 °C in a column oven and the temperature of the reactor was 135 °C. For the mobile phase, a buffer set (PF-1, PF-2, PF-3, PF-4, PF-6, PF-RG, R-3 and C1, Kanto Co., Tokyo, Japan) was used with a flow rate of 1 mL/min. Each free amino acid was identified by comparing the retention time with that of amino acids mixture standard solution type AN-Ⅱ and B (FUJIFILM Wako Pure Chemical Co., Osaka, Japan).

### 2.5. Identification of Phenolic Compounds in FP

To prepare the sample for HPLC analysis, 10 mL of FP was filtered with 0.45 μm PVDF syringe filter and evaporated at 37 °C. The FP concentrates were diluted with DW at 20 mg/mL. The phenolic compounds in FP were identified by HPLC-PDA (Waters Co., Milford, MA, USA). After 40 μL of sample injection, separation was carried out with a Phenomenex Luna C18 column (250 × 4.6 m^2^, 5 μm). The separation process was performed in a ternary mobile phase gradient (solvent A, 0.1% trifluoroacetic acid in water; solvent B, acetonitrile) at a flow rate of 1 mL/min. The composition of solvent A was maintained at 90% for 10 min and gradually decreased to 0% for 90 min.

### 2.6. Experimental Design

**Figure 1:**
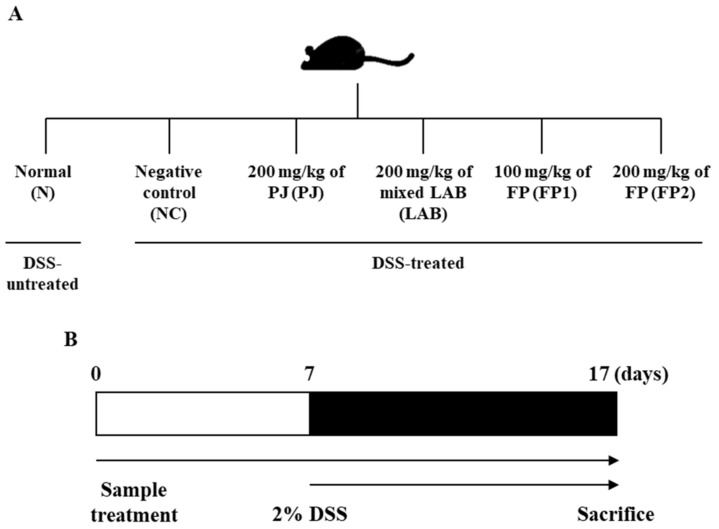
Scheme of the in vivo experimental design. (**A**) Separation of groups (*n* = 6) and (**B**) the schedule of dextran sodium sulfate (DSS) and sample treatment. 2% DSS was treated to induce colitis without N group from days 0 to 7, then samples were treated for 10 days from days 7 to 17. Serum and organs were collected on day 17 after sacrifice.

Four-week-old male ICR mice were purchased from Hyo-Chang Science Inc. (Busan, Korea). The mice were housed in an acryl cage at 22 ± 2 °C with free access to diet and water. All experimental animals were fed pellets of commercial chow for the experimental period. After a week of acclimation, the mice were randomly divided into six groups (*n* = 6): normal control group, N; DSS-administered negative control group, NC; supplemented with DSS and 200 mg/kg of PJ (2.5%, *w*/*v*) group, PJ; supplemented with DSS and 200 mg/kg of co-cultivated with *L. plantarum* and *L. casei* group (approximately 10^9^ CFU/mL), LAB; supplemented with DSS and 100 mg/kg of FP group, FP1; supplemented with DSS and 200 mg/kg of FP group, FP2 (Figure 1A). During the in vivo experiment, 2% of DSS was administered for induction of colitis. Samples were administered with mice from days 0 to 7 (Figure 1B). At the end of the experimental period, mice were sacrificed with CO_2_ gas and blood samples were collected from the inferior vena cava and placed at room temperature for 1 h, and then centrifuged at 2500× *g* for 20 min to get the serum samples. The distal portion of the colon was removed and rinsed with saline. All samples were stored at −80 °C in a deep freezer. All experimental animals were strictly treated according to the Dong-A University guidelines for the care and use of laboratory animals (DIACUC-18-2).

### 2.7. Lipid Peroxidation

Lipid peroxidation was investigated by thiobarbituric acid reactive substances (TBARS) assay by the method of Hartmann et al. (2014) [24] with some modifications. The malondialdehyde (MDA) was evaluated by 0.1g of liver homogenates in phosphate buffered saline. After homogenization, samples were centrifuged at 3500× *g* at 4 °C for 10 min, then, the supernatants were used for study. An amount of 1 mL of samples were mixed with 2 mL of thiobarbituric acid (TBA) and heated at 100 °C for 15 min and cooled on room temperature. The reacted samples were centrifuged at 3000 rpm for 10 min and the absorbance was determined using a microplate reader (Molecular Devices, Inc., San Jose, CA, USA) at 535 nm.

### 2.8. Hematoxylin and Eosin (H&E) Staining Assay

Histopathological analysis in colon tissue was conducted by the method described by Fischer et al. (2008) [25]. The colon samples were fixed by 4% formaldehyde solution and cut into 4 μm sections, and then stained with H&E solutions. The slides were observed using a light microscopy at ×100 magnification.

### 2.9. Evaluation of Inflammatory Cytokines

To analyze inflammatory cytokines, 0.1 g of colon tissues were homogenized with 1 mL of PBS in a glass tube on ice. Then, homogenates were centrifuged for 10 min at 5000× *g* to get the supernatant as samples. Serum samples were gathered by blood collected from inferior vena cava with centrifuged at 2500× *g* for 20 min. The levels of TNF-α, IFN-γ, IL-1β, IL-6, IL-12 and IL-17 in serum and colon tissues were evaluated using commercial ELISA kits according to the manufacturer’s protocol (Elabscience Biotechnology Inc., Houston, TX, USA).

### 2.10. Determination of Apoptosis in Colon Tissue

The paraffin-embedded colon tissue was cut into 4 μm. After deparaffinization, the slides were covered with the 0.3% of hydrogen peroxide diluted with distilled water (DW) to block endogenous peroxidase for 15 min at room temperature and rinsed with DW. The slides were reacted with proteinase K solution for 20 min at 37 °C. Afterwards, the terminal deoxynucleotidyl transferase solution and label solution were added to the slides. DAB solution and Mayer hematoxylin was used for development and counter stain, then dehydrated by the automated system. At least three random fields of the colonic tissues were photographed per section at ×200 magnification. The TUNEL-positive area was calculated by ImageJ software (NIH).

### 2.11. Statistical Analysis

All data are analyzed as the means ± S.D. Data were evaluated by one-way analysis of variance (ANOVA) and the statistical significances were determined using Duncan’s multiple-range test at *p* < 0.05.

## 3. Results and Discussion

### 3.1. Changes in the Total Soluble Solids, pH and Cell Count

**Figure 2:**
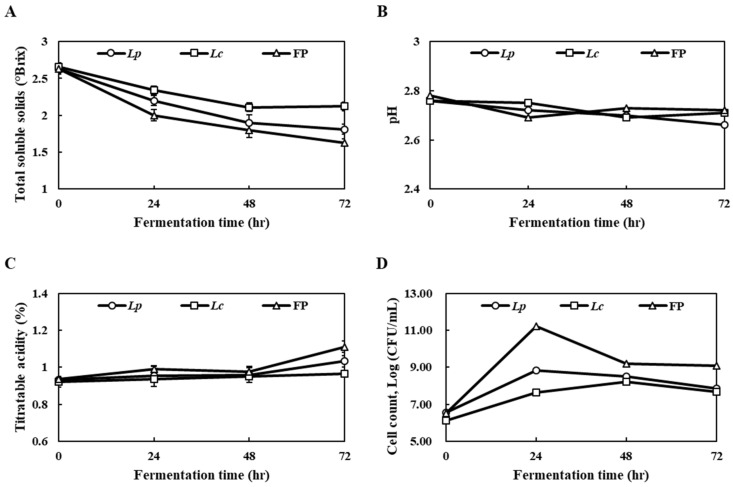
Changes of the physicochemical properties; (**A**) total soluble solids, (**B**) pH, (**C**) titratable acidity and (**D**) viable *Lactobacillus* count in *Prunus mume* juice during fermentation with mono- and mixed cultures of *L. plantarum* and *L. casei. Lp,* fermented *Prunus mume* juice with *L. plantarum*; *Lc*, fermented *Prunus mume* juice with *L. casei*; FP, fermented *Prunus mume* juice with *L. plantarum* and *L. casei*. Data are mean ± S.D. of three independent measurements.

The physicochemical properties of the fermented *Prunus mume* juice with *L. plantarum* (*Lp*), fermented *Prunus mume* juice with *L. casei* (*Lc*) and FP were investigated every 24 h. At the end of fermentation, the total soluble solids of the samples, which ranged from 1.63 to 2.12 °Brix, suggested that FP co-cultivated with *L. plantarum* and *L. casei* showed the lowest total soluble solids (Figure 2A). The changes in the pH of samples were observed from pH 2.66 to 2.72, indicating slight decreases compared to the initial value (Figure 2B). The titratable acidity of each sample, in terms of lactic acid, showed significant increases, particularly with FP from the initial titratable acidity of 0.94% to 1.11% at the end of fermentation (Figure 2C). On the other hand, there were few changes in the titratable acidity on *Lp* and *Lc* during fermentation. The FP showed significant increases in the viable *lactobacillus* cell count, indicating 11.22 log CFU/mL at 24 h of fermentation, but the viable cell count levels had decreased at 48 h and 72 h fermentation (Figure 2D). In a previous study, Lizardo et al. (2020) [18] reported that mixed LAB-fermented cherry silverberry extract showed the effective production of viable *lactobacillus* compared to mono LAB-fermentation under harsh conditions such as acidity and low pH. In addition, the pH decreased and the cell counts increased during the LAB-fermentation of kombucha [26]. Mixed-fermentation with LAB strains using food materials contributed to higher survival rates than mono-fermented cells. Therefore, new opportunities for the functionality of the product and resource utilization are expected [23]. Overall, these results suggest that the number of viable cells from mixed-fermentation with *L. plantarum* and *L. casei* was increased using the nutrients included in PJ and might be responsible for the functional properties of FP.

### 3.2. Contents of Organic Acids, Free Amino Acids and Phenolic Compounds of FP

**Figure 3:**
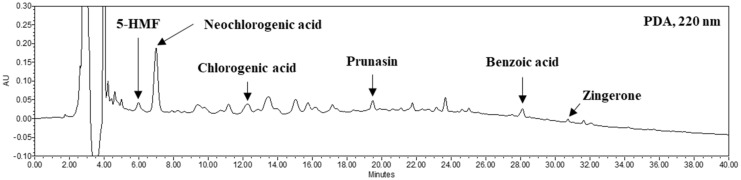
Phenolic compounds in FP analyzed by HPLC. 5-HMF (5.917 min); neochlorogenic acid (6.988 min); chlorogenic acid (12.244 min); prunasin (19.466 min); benzoic acid (28.093 min); zingerone (30.711 min).

*Prunus mume* contains various organic acids, free amino acids and phenolic compounds in fresh and pomace [8,27,28]. The organic acids of FP contained 739.81 mg% of citric acid, 184.27 mg% of lactic acid and 71.32 mg% of acetic acid (Table 2). FP contains 33.32 ppm, 4.35 ppm, 3.39 ppm and 2.29 ppm of free amino acids, aspartic acid, glutamic acid, alanine and taurine, respectively. To identify the phenolic compounds in FP, HPLC analysis was carried out. The 5-HMF, neochlorogenic acid, chlorogenic acid, prunasin, benzoic acid and zingerone with concentrations of 0.65, 5.18, 0.99, 0.52, 0.56 and 0.11 mg/g, respectively, were identified by comparison with each standard phenolic acid (Figure 3). In a previous study, PJ contained a high level of organic acids such as citric acid (969.23 mg%) and malic acid (352.83 mg%) and free amino acids such as aspartic acid (19.66 ppm), tyrosine (1.12 ppm) and phenylalanine (0.33 ppm) [8]. Chidi et al. (2018) [29] reported changes in organic acids during fermentation. Specifically, the malic acid contents included in the fruit could be converted to lactic acid. In the previous study, the organic acids contents of PJ showed high increases of acetic acid, succinic acid and lactic acid after two-step fermentation for production of *Prunus mume* vinegar as a result of the metabolism of microorganisms [8]. Mazzoli et al. (2014) [17] reported that LAB on sugar fermentation produce metabolites such as lactic acid and acetic acid on their metabolic pathways. Protein degradation of the substrate during fermentation might induce changes in the free amino acid content resulting from the metabolism of microorganisms [30,31]. Adebo et al. (2020) reported the impact of fermentation on whole grains of which increased phenolics and antioxidant properties [32]. Zhao et al. (2016) reported that the phenolic compounds in tea extracts were modified by fermentation with LAB and increased antioxidant activity and cellular uptake of phenolic compounds on Caco-2 cells [33]. Therefore, these results suggest that the compositions of organic acids, free amino acids and phenolic compounds in FP were slightly different from those in the PJ and the protective effect of FP could be enhanced.

### 3.3. Effect of FP on the Colon Length and Lipid Peroxidation

**Figure 4:**
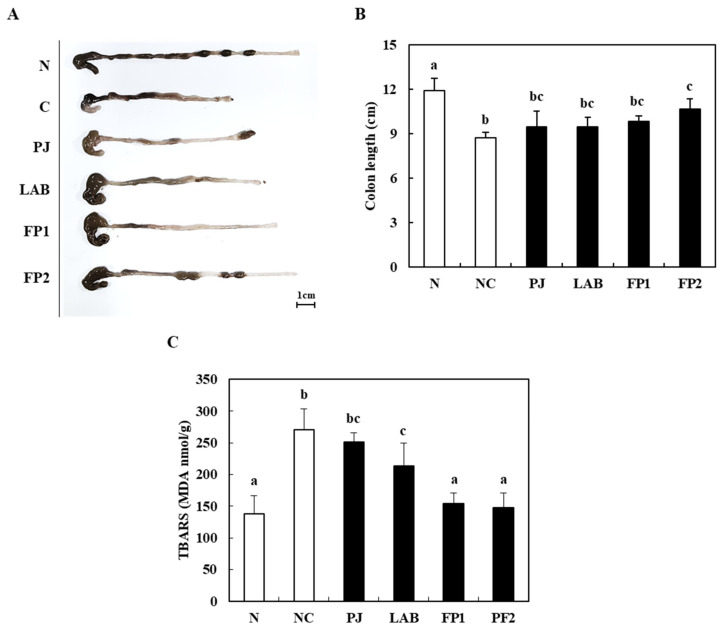
Colon length and lipid peroxidation after supplementation of FP to DSS-induced colitis mice. (**A**,**B**) Representative photos and length of the colons of the normal group and DSS-induced colitis mice supplemented with and without FP compared to the DSS control group. (**C**) Malondialdehyde contents in liver of the normal group and DSS-induced colitis mice supplemented with and without FP compared to the DSS control group. N, normal group, non-DSS treatment; NC, negative control group, DSS treatment and supplementation with distilled water; PJ; *Prunus mume* juice (PJ) group, DSS treatment and supplementation with 200 mg/kg of 2.5% PJ; LAB, lactic acid bacteria (LAB) group, DSS treatment and supplementation with 200 mg/kg of *L. plantarum* and *L. casei* suspended by distilled water; FP1, low dose of fermented *Prunus mume* juice (FP) group, DSS treatment and supplementation with 100 mg/kg of FP; FP2, high dose of FP group, DSS treatment and supplementation with 200 mg/kg of FP. Scale bars: 1 cm. Data values are expressed as the means ± S.D. (*n* = 6). The data are analyzed with one-way ANOVA and different letters on the bar show the difference in Duncan’s multiple range test (*p* < 0.05).

Figure 4 shows the changes in colon length, body weight and malondialdehyde (MDA) in DSS-treated mice. The colon length of the control group treated with DSS showed a significantly shortened large intestine length compared to the normal group (8.70 cm). In contrast, the high dose of FP (200 mg/kg)-supplemented group (FP2) showed a significantly longer colon length (11.89 cm) (Figure 4B). MDA is one of the inflammatory biomarkers related to the lipid peroxidation of tissues induced by oxidative stress, producing inflammatory markers in the damaged tissues [8,34]. Compared to the normal group, the MDA level of DSS-induced colitis mice increased significantly in the control group. The oral administration of FP1 and FP2 showed a remarkable decrease in the MDA level compared to the control group (Figure 4C). At the end of the experiment, the body weight (BW) of the normal group was 36.26 g and the BW of the control group with colitis induced by a DSS treatment was 33.61 g (Table 3). In previous studies, DSS-induced colitis usually causes shortening of the colon length, weight loss and histological damage to the colorectal tissues in mice [35]. Moreover, the supplementation of *Boswellia serrata* extract with high antioxidant activity had a protective effect on the experimental colitis model [24]. This suggests that the supplementation of FP alleviated the symptoms of colitis induced by a DSS treatment.

### 3.4. Effects of FP on Colonic Tissue Damage

**Figure 5:**
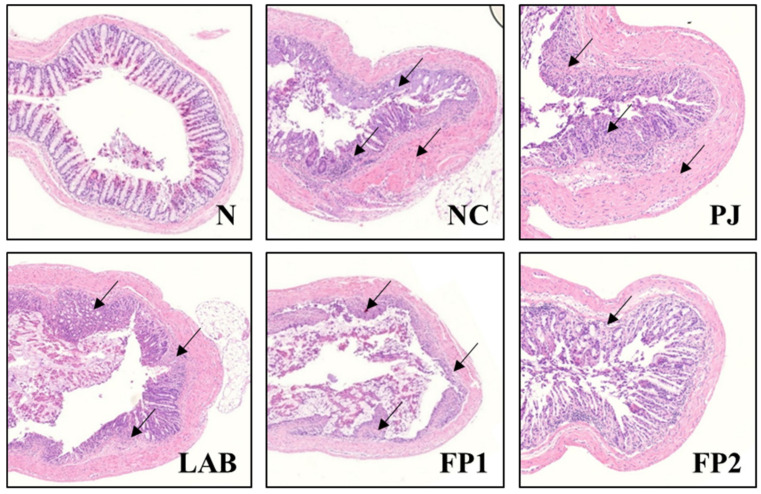
Effects of FP on H&E-stained histological changes in colonic tissue to DSS-induced colitis mice. Arrows indicate damages on colonic tissues such as inflammatory cell infiltrates and epithelial erosions. Magnification: ×100. N, normal group, non-DSS treatment; NC, negative control group, DSS treatment and supplementation with distilled water; PJ; *Prunus mume* juice (PJ) group, DSS treatment and supplementation with 200 mg/kg of 2.5% PJ; LAB, lactic acid bacteria (LAB) group, DSS treatment and supplementation with 200 mg/kg of *L. plantarum* and *L. casei* suspended by distilled water; FP1, low dose of fermented *Prunus mume* juice (FP) group, DSS treatment and supplementation with 100 mg/kg of FP; FP2, high dose of FP group, DSS treatment and supplementation with 200 mg/kg of FP.

DSS is toxic to colonic tissues and induces erosions on the epithelium, ultimately impairing the barrier integrity by increasing the colonic epithelial permeability [36]. An H&E staining assay was conducted to investigate the histological changes to the colon of DSS-administered colitis mice (Figure 5). As expected, the clear mucosa and crypt were captured in the normal group. In the DSS-induced colitis negative control group, severe damage was observed with the typical symptoms of colitis e.g., depletion of mucin, epithelial injury and ulceration. In contrast, the colon tissue of the FP-supplemented group showed increased restoration in crypt construction and epithelial erosions compared to the NC group. The supplementation of LAB-fermented rice bran alleviated the symptoms of DSS-induced colitis in mice by increasing the BW and colon length, diarrhea and inflammatory cell infiltration [37]. A *Prunus mume* extract had a therapeutic effect with the restoration of the crypt in colitis lesions on a DSS-administered colitis mice model [13]. This result suggests that FP administration alleviated the DSS-induced histopathological damages besides shortened colon length, loss of BW and increased lipid peroxidation in previous results.

### 3.5. Effects of FP on the Serum Inflammatory Cytokines

**Figure 6:**
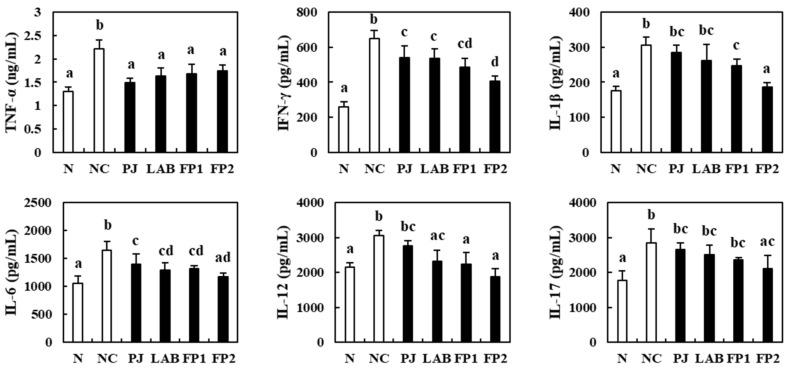
Effects of FP on serum cytokines of DSS-induced colitis mice. N, normal group, non-DSS treatment; NC, negative control group, DSS treatment and supplementation with distilled water; PJ; *Prunus* mume juice (PJ) group, DSS treatment and supplementation with 200 mg/kg of 2.5% PJ; LAB, lactic acid bacteria (LAB) group, DSS treatment and supplementation with 200 mg/kg of *L. plantarum* and *L. casei* suspended by distilled water; FP1, low dose of fermented *Prunus mume* juice (FP) group, DSS treatment and supplementation with 100 mg/kg of FP; FP2, high dose of FP group, DSS treatment and supplementation with 200 mg/kg of FP. Data values are expressed as the means ± S.D. (*n* = 6). The data are analyzed with one-way ANOVA and different letters on the bar show the difference in Duncan’s multiple range test (*p* < 0.05).

Cytokines such as interleukin (IL), tumor necrosis factor (TNF) and interferon (IFN) are regulators of infection and inflammation [38]. For that reason, many studies related to the inhibition of pro-inflammatory cytokines have been conducted to overcome the inflammation and inflammatory diseases in the human body. This study evaluated the influence of FP on serum pro-inflammatory cytokines using ELISA kits (Figure 6). The pro-inflammatory cytokines in the serum of the DSS-administered negative control group were significantly higher than the normal group, with significant incretion in TNF-α, IFN-γ, IL-1β, IL-6, IL-12 and IL-17. Compared to the NC group, the PJ supplemented group showed decreases in the pro-inflammatory cytokines, but there were no significant differences observed except for IL-1β, IL-12 and IL-17. The LAB group showed a significant decrease in TNF-α, IFN-γ, IL-6 and IL-12. As expected, supplementation of 100 and 200 mg/kg of FP decreased TNF-α, IFN-γ, IL-1β, IL-6 and IL-12 significantly in DSS-treated mice. Compared to the NC group, the high dose of FP (200 mg/kg) supplemented group showed decreases in IFN-γ, IL-1β, IL-6, IL-12 and IL-17 to 37.81%, 38.99%, 28.46%, 38.18% and 25.58%, respectively. In a previous study, supplementation with *Crytocarpa procera* bark extract attenuated the colonic mucosal damage and decreased the serum IFN-γ and IL-1β levels in DSS-induced colitis mice [39]. Supplementation with *L. fermentum* CQPC04 ameliorated the myeloperoxidase activity and pro-inflammatory cytokines e.g., TNF-α, IFN-γ, IL-1β, IL-6 and IL-12 in the experimental colitis model [40]. These findings suggest that the administration of FP alleviated inflammation by down-regulating the level of serum pro-inflammatory cytokines in DSS-induced colitis mice.

### 3.6. Effects of FP on Colonic Inflammatory Cytokines

**Figure 7:**
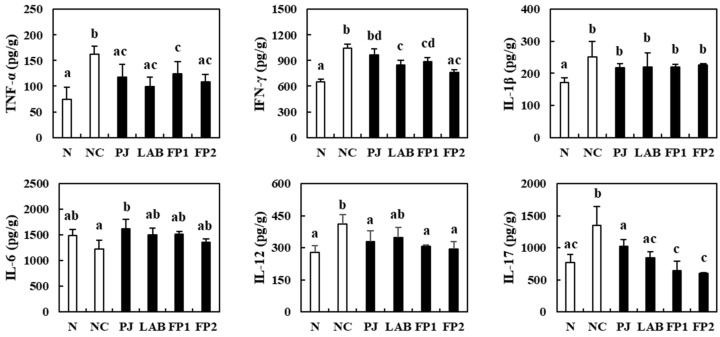
Effects of FP on colonic cytokines of DSS-induced colitis mice. N, normal group, non-DSS treatment; NC, negative control group, DSS treatment and supplementation with distilled water; PJ; *Prunus* mume juice (PJ) group, DSS treatment and supplementation with 200 mg/kg of 2.5% PJ; LAB, lactic acid bacteria (LAB) group, DSS treatment and supplementation with 200 mg/kg of *L. plantarum* and *L. casei* suspended by distilled water; FP1, low dose of fermented *Prunus mume* juice (FP) group, DSS treatment and supplementation with 100 mg/kg of FP; FP2, high dose of FP group, DSS treatment and supplementation with 200 mg/kg of FP. Data values are expressed as the means ± S.D. (*n* = 6). The data are analyzed with one-way ANOVA and different letters on the bar show the difference in Duncan’s multiple range test (*p* < 0.05).

The changes of inflammatory cytokines in the colonic tissue of DSS-induced colitis mice model were evaluated further (Figure 7). The negative control group, the DSS-administered group, showed significantly higher levels of TNF-α, IFN-γ, IL-1β, IL-12 and IL-17 than the normal group. In contrast, FP supplementation decreased the levels of TNF-α, IFN-γ, IL-12 and IL-17 significantly compared to the NC group. The supplementation of PJ significantly decreased the levels of TNF-α, IL-12 and IL-17. The LAB group showed significant decreases of TNF-α, IFN-γ and IL-17. Many studies on the supplementation of *Lactobacillus* strains have focused on ameliorating colitis by regulating cytokines and alleviating tissue damages. *L. casei* protected mucosa damage and modulated the immune response against intestinal inflammation in DSS-treated mice and *L. plantarum* increased the induction of regulatory T cells and type 2 helper T cells in the spleen and suppressed pro-inflammatory cytokines e.g., TNF-α, IL-17 and IL-1β [41,42]. In addition, LAB-fermented bread showed high levels of phenolics and inhibition of the secretion of pro-inflammatory cytokines in vitro [43]. The functions of LAB could be multiplied with co-cultures, particularly in utilization with food [23]. This suggests that the synergistic effects of mixed fermentation with *Lactobacillus* strains and PJ decrease the levels of pro-inflammatory cytokines in the serum and colonic tissue in the FP-supplemented group.

### 3.7. Effects of FP on Apoptosis in Colonic Tissue

**Figure 8:**
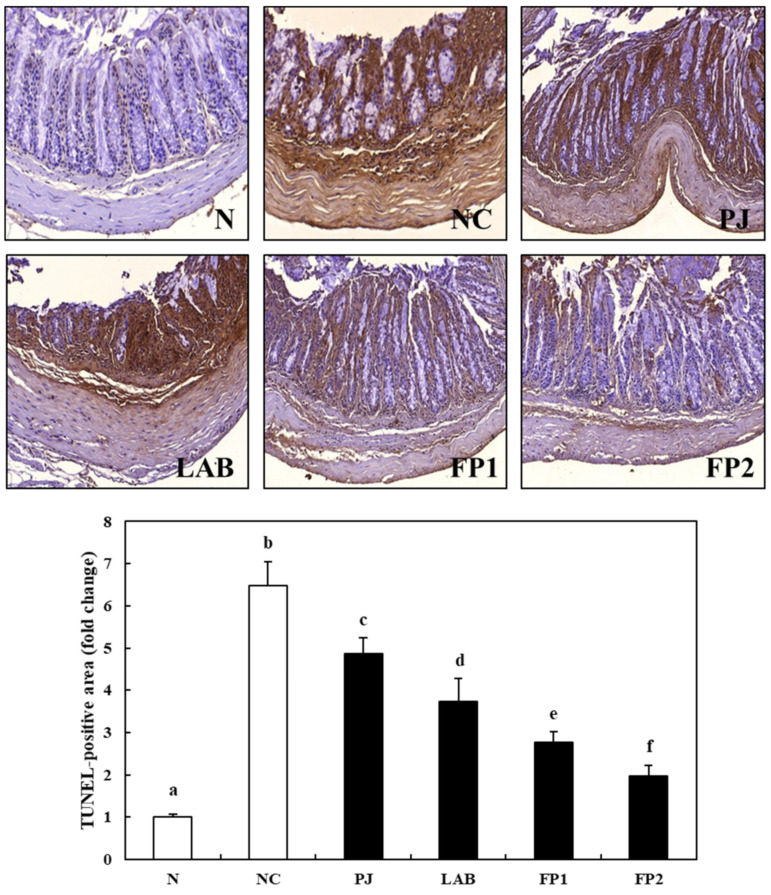
Effects of FP on TUNEL stained cell apoptosis in colonic tissue to DSS-induced colitis mice. Magnification: ×200. N, normal group, non-DSS treatment; NC, negative control group, DSS treatment and supplementation with distilled water; PJ; *Prunus mume* juice (PJ) group, DSS treatment and supplementation with 200 mg/kg of 2.5% PJ; LAB, lactic acid bacteria (LAB) group, DSS treatment and supplementation with 200 mg/kg of *L. plantarum* and *L. casei* suspended by distilled water; FP1, low dose of fermented *Prunus mume* juice (FP) group, DSS treatment and supplementation with 100 mg/kg of FP; FP2, high dose of FP group, DSS treatment and supplementation with 200 mg/kg of FP. Data values are expressed as the means ± S.D. (*n* = 6). The data are analyzed with one-way ANOVA and different letters on the bar show the difference in Duncan’s multiple range test (*p* < 0.05).

Apoptosis, one of the major points on the diagnosis of IBD, is called programmed cell death, which occurs mainly in histopathological studies in colitis lesions, accelerating the depletion of the mucosal barrier and inflammatory erosions, and the invasion of bacterial pathogens [44,45]. A TUNEL assay was carried out to evaluate the effect of FP on inhibition of apoptosis in colon tissue. The TUNEL-positive cells appeared as a brown color and normal cells appeared as a blue color. As shown in Figure 8, the TUNEL-positive cells were commonly observed in the NC group indicating the induction of apoptosis by DSS supplementation. In addition, the number of apoptotic cells in the colon tissue of the PJ and LAB group, which were identified from the levels of the TUNEL-positive area, was significantly lower than in the NC group. In the contrast, the TUNEL-positive cells decreased significantly according to the supplementation of 100 mg/kg and 200 mg/kg of FP. Shin et al. (2017) [46] reported that rosuvastatin inhibited the induction of apoptosis activated by the pro-inflammatory cytokines secreted in the DSS-induced colitis model. Chae et al. (2019) [47] reported that the supplementation of the probiotic lactic acid bacteria, *L. plantarum* LB-9, ameliorated colitis by inhibiting the TNF-α mediated apoptosis of intestinal epithelial cells in the DSS-induced colitis mice model. Further studies will be required to probe the effects of FP on the molecular mechanisms related to the inflammatory cytokines and apoptosis. Nevertheless, based on these results, FP supplementation inhibited the apoptosis of intestinal epithelial cells activated by pro-inflammatory cytokines in DSS-induced colitis mice.

## 4. Conclusions

FP containing diverse free amino acids and organic acids was produced by mixed fermentation with PJ and two strains of LAB: *L. plantarum* and *L. casei*. FP supplementation prevented the typical symptoms of colitis, such as loss of BW, shortened colon length, lipid peroxidation and colonic damages. Furthermore, it attenuated the increased pro-inflammatory cytokines in the serum and colon in a DSS-induced colitis mice model. FP administration also inhibited the apoptosis caused by the activation of secreted pro-inflammatory cytokines in colon tissue. Overall, FP can be utilized as a potential source against the prevention of colitis.

## Figures and Tables

**Table 1:** *Prunus mume* juice (PJ) fermentation with lactic acid bacteria (LAB).

Strains	*Lp*	*Lc*	FP
*Lactobacillus plantarum*	+		+
*Lactobacillus casei*		+	+

**Table 2:** Contents of organic acids and free amino acids in FP.

Organic Acids	Contents (mg%)	Free Amino Acids	Contents (ppm)
Citric acid	739.81 ± 17.61	Aspartic acid	33.32 ± 2.21
Lactic acid	184.27 ± 11.43	Glutamic acid	4.35 ± 0.74
Acetic acid	71.32 ± 4.52	Alanine	3.39 ± 0.35
Malic acid	N.D.	Taurine	2.29 ± 0.27
Total organic acids	996.07 ± 58.82	Total free amino acid	66.43 ± 4.25

Data values are expressed as the means ± S.D. (*n* = 3). N.D.; not detected.

**Table 3:** Changes of body weight after in vivo experiment.

Body-Weight (g)	N	NC	PJ	LAB	FP1	FP2
Initial	33.94 ± 0.81	34.11 ± 0.68	34.72 ± 1.04	34.71 ± 0.95	33.97 ± 1.65	34.61 ± 1.15
Final	36.26 ± 1.83	33.61 ± 1.82	34.69 ± 3.31	35.14 ± 2.32	34.59 ± 3.58	35.61 ± 2.52

Data values are expressed as the means ± S.D. (*n* = 6).

## Data Availability

Data is contained within the article.
